# Mediators in Psychological Treatments for Anxiety and Depression in Adolescents and Young People: A Protocol of a Systematic Review

**DOI:** 10.3389/fpsyg.2021.708436

**Published:** 2021-07-21

**Authors:** Sonia Conejo-Cerón, Svenja Taubner, Erkki Heinonen, Asta Adler, Rasa Barkauskiene, Dina Di Giacomo, Yianna Ioannou, Jose M. Mestre, Margarida Rangel Henriques, Catarina Pinheiro Mota, Sonja Protić, Marija Raleva, Filipa Mucha Vieira, Jan Ivar Røssberg, Célia M. D. Sales, Andrea Saliba, Stefanie J. Schmidt, Tjaša Stepišnik Perdih, Randi Ulberg, Jana Volkert, Patricia Moreno-Peral

**Affiliations:** ^1^Instituto de Investigación Biomédica de Málaga, Málaga, Spain; ^2^Institute for Psychosocial Prevention, University Heidelberg, Heidelberg, Germany; ^3^National Institute for Health and Welfare, Helsinki, Finland; ^4^Institute of Psychology, Vilnius University, Vilnius, Lithuania; ^5^Department of Life, Health and Environmental Sciences, University of L’Aquila, L’Aquila, Italy; ^6^Department of Social Sciences, University of Nicosia, Nicosia, Cyprus; ^7^Department of Psychology, Universidad de Cádiz, Cádiz, Spain; ^8^Faculty of Psychology and Education Science, University of Porto, Porto, Portugal; ^9^Center for Psychology at University of Porto, Porto, Portugal; ^10^Departamento de Educação e Psicologia, University of Trás-os-Montes and Alto Douro, Vila Real, Portugal; ^11^Institute of Criminological and Sociological Research, Belgrade, Serbia; ^12^Department of Child and Adolescent Psychiatry, University Clinic Skopje, Skopje, Macedonia; ^13^Institute of Clinical Medicine, University of Oslo, Oslo, Norway; ^14^Psychiatric Research Unit, Division of Mental Health and Addiction, Oslo University Hospital, Oslo, Norway; ^15^University of Malta, Mental Health Services Malta, Msida, Malta; ^16^Department of Clinical Psychology and Psychotherapy, University of Bern, Bern, Switzerland; ^17^School of Advanced Social Studies, Nova Gorica, Slovenia; ^18^Department of Psychiatry, Diakonhjemmet Hospital, Oslo, Norway; ^19^MSB Medical School Berlin, Berlin, Germany

**Keywords:** systematic review, anxiety, depression, young adult, adolescence, mediator, psychotherapy

## Abstract

**Introduction:**

Anxiety and depressive disorders are a significant problem that starts in childhood or adolescence and should be addressed early to avoid chronic mental conditions. There is strong evidence to demonstrate that psychological treatments are effective for these disorders, however, little is known on mediators and mechanisms of change of psychological treatment in adolescents and young adults. Understanding the pathways through which psychological treatments operate will facilitate more effective treatments.

**Aim:**

We aim to conduct a systematic review, exploring the available evidence on mediators of psychological treatments for anxiety and depression in adolescents and young adults.

**Methods:**

A systematic search has been performed on PubMed and PsycINFO databases to identify studies from inception to 23rd February 2020. Eligible studies include randomized controlled trials and trials (quasi-experimental) designs that have enrolled adolescents and young adults presenting with depression and/or anxiety and that have examined mediators of psychological treatments. A group of 20 reviewers from the COST-Action TREATme (CA16102) divided into 10 pairs independently screen studies for inclusion, extract information from the included studies, and assess the methodological quality of the included studies and the requirements for mediators. The methodological quality will be assessed by The Mixed Methods Appraisal Tool. Extracted data from the included studies will be collected and presented using a narrative approach.

**Discussion:**

This systematic review will summarize and provide a comprehensive overview of the current evidence on mediators of psychological treatments for anxiety and depression for adolescents and young adults. Results will allow the identification of strategies to optimize intervention to enhance clinical outcomes.

**Ethics and dissemination:**

Ethics approval is not required. Findings from this systematic review will be published in a peer-reviewed journal and disseminated at conferences and meetings. PROSPERO registration number: CRD42021234641.

## Introduction

Anxiety and depressive disorders are a significant public health concern. According to the World Health Organization (WHO), approximately 264 and 322 million people suffer from anxiety and depressive disorders, respectively (World Health Organization, 2017). Comorbidity between anxiety and depressive disorders is highly common and the risk of one disorder can increase the risk of another (Kessler et al., 2011; Cummings et al., 2014). In terms of disease burden, anxiety and depressive disorders are among the leading causes of years lived with disability for all ages (GBD, 2020). Both disorders are associated with high economic costs (Olesen et al., 2012) and depression is associated with high mortality (Cuijpers et al., 2014).

Adolescents and young adults are a fundamental and vulnerable group with distinct mental health needs. Anxiety disorders typically begin in childhood, the median age of onset being 11 years, whereas depression frequently manifests later during adolescence or early adulthood, and its mean age of onset has been estimated around 30 years (Kessler et al., 2005). At a global level, in 2019, anxiety and depressive disorders have been the sixth and the fourth leading cause of illness and disability among adolescents and young adults aged 10–24 years, respectively (GBD, 2020). The consequences of not addressing these emotional disorders during this period leads to considerable suffering and impaired functioning, affecting physical and mental health and limiting opportunities to lead fulfilling lives as adults limiting opportunities extend into adulthood (World Health Organization, 2020).

There is strong evidence on the effectiveness of psychological treatments for anxiety disorders (Zhou et al., 2019) and depression (Zhou et al., 2015) in children and adolescents. Cognitive-behavioral therapy (CBT) is the most researched and commonly used psychological treatment for anxiety and depressive disorders in children and adolescents (David-Ferdon and Kaslow, 2008; Silverman et al., 2008; Weersing et al., 2017). Interpersonal psychotherapy (IPT) is also considered evidence-based psychotherapy for youth depression (Birmaher et al., 2007; National Institute for Clinical Excellence, 2015; Zhou et al., 2015). IPT is less studied than CBT but shows promising results for anxiety disorders (although with no superiority compared to other bona fide therapies) (Markowitz et al., 2014). Other treatment approaches such as psychodynamic psychotherapy, acceptance and commitment therapy, or mindfulness have also been used for anxiety and depressive disorders (Abbass et al., 2013; Chi et al., 2018; González-Valero et al., 2019; Harris and Samuel, 2020; Midgley et al., 2021).

However, the treatment effect sizes have substantial room for improvement. A meta-analysis of the youth therapy evidence base conducted by Weisz et al. (2017) found a medium effect size for treating anxiety when they compared active treatments vs. control condition. In depression, the differences between-group treatment effects were smaller, showing small to medium effect sizes (Weisz et al., 2017; Eckshtain et al., 2020). IPT has shown greater effect sizes than other psychological treatments for depression in the treatment of adolescents, although the number of RCTs that evaluated the effectiveness of IPT was much more limited (Eckshtain et al., 2020). In the case of anxiety, the evidence for the effectiveness of IPT is very scarce (Markowitz et al., 2014). González-Valero et al. (2019) performed a meta-analysis of the effects of mindfulness-based approaches, self-reflection and cognitive behavioral therapy in youth showing satisfactory and significant results in relation to the reduction of anxiety and depression in youth. Another meta-analysis focused exclusively on mindfulness had moderate effects in reducing depression in young people at post-test (Chi et al., 2018). Regarding psychodynamic therapy, the evidence suggests this approach may be especially effective for treating anxiety and depression in children and adolescents (Abbass et al., 2013; Midgley et al., 2021). In order to optimize treatments, one of the main challenges for psychotherapy research is to identify the mechanisms and therapeutic processes that lead to positive outcomes and improvements over the course of psychological treatments. Mechanisms of change define causal relationships between psychological treatments and therapeutic change. A mechanism of change explains how a treatment translates into a process that leads to an outcome (Kazdin, 2007). Understanding the mechanisms through which psychological treatments operate will likely facilitate the development of new treatments with better outcomes and, possibly, greater cost-effectiveness. In this way, the active therapeutic components could be intensified and refined, while the inactive or redundant elements could be discarded (Kazdin and Weisz, 1998; Kraemer et al., 2002).

An important first step toward examining mechanisms of change in psychological treatments is the identification of mediators of outcome (Kraemer et al., 2002; Kazdin and Nock, 2003). A mediator is a construct that shows statistical relations between treatment and outcome but may not explain the precise process through which change comes about (Kazdin, 2007). Kazdin’s (2007) recommendations to better understand the mediators and mechanisms of therapy are the following: (1) use theory of psychological change as a guide, (2) include measures of potential mediators in treatment studies, (3) establish the timeline of the proposed mediator or mechanism and outcome, (4) assess more than one mediator or mechanism, (5) use designs that can evaluate mediators and mechanisms (randomized controlled trials -RCTs- are excellent designs in demonstrating a causal relationship between the treatment and therapeutic change), (6) examine consistencies across different types of studies, and (7) intervene to change the proposed mediator or mechanism. Despite the recommendations on how to evaluate the mediators and mechanisms of change in psychological treatments, little progress has been made in the research on mechanisms of change in the treatment of adolescents and young adults (Kazdin and Nock, 2003). Cuijpers et al. (2019) concluded that we have no empirically validated mechanisms of change in adult psychotherapy after several decades of systematic psychotherapy research.

Some efforts have been made to identify mediators of psychological treatments in the treatment of young adults with depression. The reviews on this topic have focused mainly on CBT and, to a lesser extent, on IPT. Weersing and Weisz (2002) conducted a systematic review which included RCTs targeting various youth problems. For depression, they identified 12 RCTs that assessed some candidate mediators (cognitive distortions, self-concept, social adjustment, pleasant activities, among others). Although some included studies found that psychological treatments changed the candidate mediator compared to control groups, most of the studies did not conduct a formal mediation test. The meta-analysis of Chu and Harrison (2007) included 14 RCTs on the effectiveness of CBT in depressive outcomes, but only three RCTs examined treatment mediators. This meta-analysis found that CBT had significant small-to-medium effects on cognitive candidate mechanisms and no significant effects on behavioral and coping mechanisms. After nearly a decade from Chu and Harrison’s review, Weersing et al. (2017) carried out a systematic review that included only RCTs where different candidate mediators of interventions for the treatment of young adult depression were tested. The mediators identified by the authors for CBT were cognitive, behavioral and motivational. However, these findings were based on only five RCTs and some failed to meet the basic requirements for identifying mediators, such as to establish temporal precedence of change. In another systematic review conducted by Lemmens et al. (2016) on mechanisms of change in psychotherapy for depression, some mediators such as rumination and worries were identified. They concluded that research is heterogeneous and unsatisfactory in many methodological respects, but also that psychotherapy might be too complex to be explained in simple models of psychological change. In their systematic review, the authors only included nine studies for the treatment of adolescents with depression. Recently, Ng et al. (2020) conducted a systematic review and selected 46 randomized trials of CBT and IPT with depressed youths; 74% measured candidate mediators, but only 17% analyzed these factors as mediators. Although four significant candidate mediators (negative cognition, family functioning, treatment expectancy, and motivation to change) emerged, findings were sparse, conflicting, and clouded by methodological issues. These studies highlight that only a minority of RCTs tested candidate mechanisms as mediators, and the vast majority assessed CBT.

For the treatment of anxiety, the evidence is even more limited. The systematic reviews that have been performed have focused on specific mediators or treatments and were not based on the young population. Smits et al. (2012) reviewed the evidence for the threat reappraisal mediation hypothesis for CBT treatment of anxiety disorders. Most of the studies identified included samples of adults who have panic disorder or social anxiety disorder. Therefore, it was not possible to examine whether threat reappraisal mediation of CBT efficacy varied across the anxiety disorders. The authors concluded that threat reappraisal is related to anxiety symptom improvement with CBT. However, they could not demonstrate that threat reappraisal causes symptom improvement in CBT. Moreover, they could not demonstrate that threat reappraisal is not a substitute for other third variables, since few studies meet most of the criteria necessary to establish causality. Another systematic review carried out by Gregory and Peters (2017) showed that change in self-related constructs (self-esteem, self-schema, self-focused attention, and self-evaluation) predicted and/or mediated social anxiety reduction. However, the studies were very few and had methodological limitations. On the other hand, Fentz et al. (2014) studied the mediational role of panic self-efficacy in CBT for panic disorder. Results provided some support for panic self-efficacy as a mediator of treatment outcome, although none of the studies met all of the criteria proposed by the authors for establishing mediation. In their meta-analysis on the effectiveness of CBT in anxiety outcomes for youth, Chu and Harrison (2007) found that CBT had statistically significant and large-sized effects on behavioral processes and moderate effects on physiological and cognitive processes and coping. However, the vast majority of the studies included in this meta-analysis did not report a formal test of mediation. Finally, two reviews summarized studies testing mediators in youth. The first review was conducted by Weersing and Weisz (2002) and identified one study where changes in arousal were related to anxiety measures, although this study did not conduct analyses to test for mediated effects. The second review, by Silverman et al. (2008), identified two studies on cognitive mediators in youth psychotherapy for anxiety. They concluded that self-talk and positive self-statements mediated change in anxiety symptoms; however, these mediators were not assessed during treatment.

Since both the presentation of psychological symptoms and psychological treatments for adolescents and young adults are slightly different from those for adults, the potential mediators and mechanisms of change may also differ between adults and youth. To our knowledge there are no systematic reviews of mediators of all branches or types of psychological treatments for anxiety and/or depression in adolescents and young adults. The aims of this systematic review will be: (1) to identify which mediators and theories of change have been studied in psychological treatments for anxiety and depression in adolescents and young adults, (2) to identify those mediators and theories of change with the strongest empirical support for the treatment of anxiety and depression in adolescents and young adults, and (3) to critically evaluate the methodological characteristics and quality of the current research data available on mediators in psychological treatments for anxiety and depression in adolescents and young adults.

This systematic review is carried out as part of the “European Network of Individualized Psychotherapy Treatment of Young People with Mental Disorders” (TREATme)^1^, funded by the European Cooperation in Science and Technology (COST). TREATme will review the academic research relating to mediators in young people receiving psychological treatments.

## Methods

### Reporting and Protocol Registration

This protocol is following the Preferred Reporting Items for Systematic Reviews and Meta-Analyses Protocols guidelines (PRISMA-P) (Moher et al., 2015) and will adhere to the PRISMA 2020 statement (Page et al., 2021). The study protocol was previously registered in the International Prospective Register of Systematic Reviews (registration number: CRD42021234641).

### Information Sources and Search Strategy

Systematic literature searches for relevant studies have been conducted in the following databases: PsycINFO and PubMed (Medline) from inception to February 23rd, 2020. The searches will be updated just before the final results are analyzed to retrieve the most recent studies for inclusion. We will perform hand-searching of the reference list of included studies and relevant systematic reviews on the topic. We will contact experts in the field to retrieve additional studies. The searches include a broad range of terms and keywords related to mediators, young people and psychological treatments. The specific search strategy used in PubMed (Medline) and PsycINFO is provided in Supplementary Material 1.

### Eligibility Criteria

Studies will be included in this systematic review based on the following criteria:

#### Participants

We will include studies involving adolescents and young adults aged between 10 and 30 years old, with a diagnosis of depression and/or anxiety through standardized instruments (e.g., Structured Clinical Interview for DSM Disorders), through validated self-reports with standard cut-off points (e.g., Beck Depression Inventory-II; Beck Anxiety Inventory-II), or diagnosis by a mental health specialist.

#### Intervention

Eligible interventions will aim at treating or ameliorating depression and/or anxiety and will include all branches or types of interventions: psychodynamic, integrative, systemic, cognitive-based or cognitive-behavioral, interpersonal, humanistic, psychoeducation, and third-wave approaches. Face-to-face interventions (individual and group), internet-based interventions (guided, unguided, psychoeducational websites) or a combination between them will be included. Interventions that are pharmacological or physical (e.g., exercise) will not be included. In addition, those studies that included adjunct pharmacotherapy or physical to a psychological treatment will also be excluded.

#### Comparator

Usual care, waiting list, attention control, or other type of comparators will be included.

#### Outcome

Studies will be included if they examine the psychological mediators and statistical analysis of mediation of psychotherapy outcome (Baron and Kenny, 1986 or more advanced methods). We will include outcome measures assessing diagnosis status and symptom severity for symptoms of anxiety and depression.

#### Study Design

Randomized controlled trials and trials (quasi-experimental) designs will be included. Other types of designs will be excluded.

#### Setting, Language, and Publication Date

Studies from any setting, written in English and published from inception onwards, will be eligible.

### Selection Procedure

A group of 20 experienced researchers (from now on reviewers), divided into 10 pairs, will conduct the study selection. Before selecting studies, the group of reviewers will develop and agree to adhere to a homogeneous screening and rating procedure. The 10 pairs of reviewers will independently assess the eligibility of studies retrieved in two phases. After duplicate studies are eliminated in the first phase, titles and abstracts of all studies retrieved will be screened. Those studies that will not meet the inclusion criteria outlined above will be excluded. In the second phase, each pair of reviewers will evaluate the full text of these potentially eligible studies to check if they meet the inclusion criteria. Any discrepancies in selected studies will be discussed in pairs, and a third reviewer will be consulted if a consensus cannot be reached. To guarantee the study selection process, independent reviewers will perform an additional quality control check by assessing the eligibility of every fifth excluded study. Discrepancies at this stage will be resolved through discussion with the original reviewer pair. A PRISMA flow chart showing the details of studies included and excluded at each phase of the study selection process will be provided.

### Data Extraction

Data extraction will also be performed independently by pairs of reviewers. Discrepancies between the reviewers will be resolved by discussion or with a third reviewer where necessary. A data extraction sheet will be used, and the following study characteristics will be extracted for each included study: study setting; study population, participant demographics, and baseline characteristics; details of the treatment and control conditions; study methodology; outcomes and times of measurement; assessed mediators; type of mediation analysis and information for the assessment of the risk of bias. We will use Microsoft Excel (2013) to manage the data extraction process.

### Data Synthesis

The characteristics of the included studies will be presented in different tables. We will synthesize the results from the included studies and draw conclusions based on the body of evidence using standard methods for narrative syntheses, as described by Popay et al. (2006). The narrative synthesis will be focused on the categories of mediators that have been tested, types of psychological treatments that have been investigated, type of population (clinical-subclinical), mental disorders or psychological symptoms (depression-anxiety) that have been treated and age range that has been considered (adolescents-young adults). Included studies can be grouped by disorder (depression-anxiety), by population (clinical-subclinical), by treatment type (e.g., cognitive behavioral therapy, interpersonal therapy) and/or age range. It will be discussed if age-, disorder- or treatment-specific mediators can be identified.

### Critical Appraisal

To evaluate the quality of the mediation studies, we will use the most relevant criteria of requirements according to Kazdin and Nock (2003); Kazdin (2007), and Lemmens et al. (2016). We will evaluate *specificity* (the mediator is specific for a particular type of therapy), *temporal relation* (the mediator should precede the outcome in time) and *experimental manipulation* (direct manipulation of the mediator through an experiment). We will use *a strong association* requirement to ascertain whether there was a statistical association between variables (Kazdin, 2007; Kazdin and Nock, 2003). According to Lemmens et al. (2016), we will also evaluate whether multiple mediators have been examined. Two reviewers will independently assess the quality of the mediation studies, and any discrepancies will be discussed until consensus is reached.

### Risk of Bias in Individual Studies

The latest version of the Mixed Method Appraisal Tool (MMAT, Hong et al., 2018) will be used to assess the quality of the studies included. The MMAT is designed to evaluate mixed studies, including five categories of studies: qualitative research, randomized controlled trials, non-randomized studies, quantitative descriptive studies, and mixed methods studies. The tool comprises two screening questions, and five criteria for each type of study scored on a categorical scale as either “yes,” “no,” or “cannot tell.” The initial two screening questions indicate whether a further methodological quality appraisal is feasible or appropriate. If responses to both questions are either “no” or “cannot tell,” they will be excluded from further evaluation. To obtain an overall quality score for each study, items score as “yes” would be summed. The overall score ranges from 0 to 5 points (“0” the lowest quality score and “5” the highest quality score). The two reviewers will independently judge the quality of the included studies, and any discrepancies will be resolved through discussion.

### Amendments to the Protocol

In case of any amendments made to this protocol when conducting the systematic review, we will document all changes in PROSPERO and the final publication.

## Discussion

This protocol lays out a plan for a systematic review to provide more knowledge about the mediators of various psychological treatments for adolescents and young adults suffering from depression and/or anxiety. Identifying likely or promising treatment mediators advances our understanding of how treatments for depression and anxiety affect adolescents and young adults. This can help develop more effective treatments and prevent treatment failure or adverse events. In addition, results might assist in the verification and refinement of how treatments for depression and anxiety might work in adolescents and young adults. Based on the results, we will have information on the similarity or difference in mediators of psychological treatments in adolescents compared to young adults.

One of the main strengths of this study is the inclusion of a large multidisciplinary group of international researchers with extensive experience in this area who have worked for 3 years on this project. Moreover, for the correct development of this protocol, the group has consulted international experts in the field. This systematic review will cover all psychological treatments and focus on the two most prevalent mental health conditions in adolescents and young adults. In addition, we will rigorously follow the PRISMA guidelines. According to the open science initiative recommendations, the data set will be made available to other research groups.

However, there could be several limitations of this study that should be considered. The substantial heterogeneity in terms of design, therapies and mediation analyses of included studies might cause one crucial limitation, which likely limits the possibility to estimate aggregated effect sizes for the identified mediators. According to Higgins and Green (2011), one of the circumstances where it may not be possible to undertake a statistical synthesis is when studies are too diverse since the results may be obscured. Although both RCTs and quasi-experimental designs are valid to demonstrate causal relationship between psychological treatments and outcomes, quasi-experimental designs have lower quality with regard to the internal validity than RCTs. According to previous studies on psychological mediators (Lemmens et al., 2016; Weersing et al., 2017; Moreno-Peral et al., 2020; Ng et al., 2020), we expect low compliance with the methodological requirements to establish as a mediator. Furthermore, only studies written in English will be included, so studies with our inclusion criteria written in other languages may not be considered. Although we plan to search for studies in two different databases, contact experts, review the reference list of included studies and relevant systematic reviews on this topic, missing some studies is inevitable.

In summary, the evidence from this systematic review will inform treatment development by highlighting the mediators responsible for therapeutic change and will extend the evidence based on the efficacy of psychological treatments for depression and anxiety in adolescents and young adults.

## Author Contributions

SC-C, ST, EH, AS, SP, JV, AA, RB, DD, YI, JM, FV, CM, MR, MH, JR, SS, TP, RU, CS, and PM-P provided a substantial contribution to the conception and design of the work by developing the research questions, the search string, and carrying out the stage 1 screening. SC-C and PM-P drafted the current manuscript. ST, EH, AS, SP, JV, AA, RB, DD, YI, JM, FV, CM, MR, MH, JR, SS, TP, RU, and CS corrected and finally approved the manuscript. RU coordinated the overall COST initiative. All authors agreed to be accountable for all aspects of the work in ensuring that questions related to the accuracy or integrity of any part of the work are appropriately investigated and resolved.

## Conflict of Interest

The authors declare that the research was conducted in the absence of any commercial or financial relationships that could be construed as a potential conflict of interest.

## Footnotes

1 www.treat-me.eu
