# Supplementary material for: Mediators in Psychological Treatments for Anxiety and Depression in Adolescents and Young People: A Protocol of a Systematic Review
Source: Front Psychol. 2021 Jul 21;12:708436. doi: 10.3389/fpsyg.2021.708436 (PMC8333613; doi:10.3389/fpsyg.2021.708436)
Supplement: Supplementary file 1 [file Table_1.DOCX]

Supplementary Material

# Supplementary Data. Search strategy.

mediat* OR mediation* OR mediating effect* OR indirect effect* OR mediator effect* OR working mechanisms OR working mechanism OR process research OR process-outcome* OR psychotherapeutic processes OR mechanism of changing OR mechanisms of changing OR mechanism of changes OR mechanisms of changes OR mechanism of change OR mechanisms of change OR psychotherapeutic mechanism OR psychotherapeutic mechanisms OR psychotherapy mechanism OR psychotherapy mechanisms OR psychotherapies mechanism OR psychotherapies mechanisms OR therapeutic mechanism OR therapeutic mechanisms OR therapy mechanism OR therapy mechanisms OR therapies mechanism OR therapies mechanisms OR process of therapy OR process of therapies OR processes of therapy OR processes of therapies OR processing of therapy OR processing of therapies OR process of psychotherapy OR process of psychotherapies OR processes of psychotherapy OR processes of psychotherapies OR processing of psychotherapy OR processing of psychotherapies OR psychotherapy process OR psychotherapies process OR psychotherapy processes OR psychotherapies processes OR psychotherapy processing OR psychotherapies processing OR therapy process OR therapies process OR therapy processing OR therapies processing OR therapy processes OR therapies processes OR psychotherapy research OR psychotherapies research OR psychotherapy researches OR psychotherapies researches OR therapy research OR therapies research OR therapy researches OR therapies researches OR psychotherapy technique OR psychotherapies technique OR psychotherapy techniques OR psychotherapies techniques OR psychotherapeutic technique OR psychotherapeutic techniques OR therapy technique OR therapies technique OR therapy techniques OR therapies techniques OR therapeutic technique OR therapeutic techniques OR psychotherapy relationship OR psychotherapies relationships OR psychotherapy relationship OR psychotherapies relationships OR psychotherapeutic relationship OR psychotherapeutic relationships OR therapy relationship OR therapy relationships OR therapies relationship OR therapies relationships OR therapeutic relationship OR therapeutic relationships OR therapy alliance OR therapy alliances OR therapies alliance OR therapies alliances OR therapeutic alliance OR therapeutic alliances OR psychotherapy alliance OR psychotherapy alliances OR psychotherapies alliance OR psychotherapies alliances OR psychotherapeutic alliance OR psychotherapeutic alliances

adolescent OR adolescent*” OR “emerging adulthood” OR “young” OR “juvenile” OR “early adulthood” OR “young adulthood” OR “young adult” OR “young adults" OR “teen*” OR “youth*” OR “yeasty” OR “juvenil*” OR “young*” OR “subadult” OR “immature” OR “adolescen*” OR “puberty” OR “pupertal” OR “puberal” OR DE "Emerging Adulthood" OR DE "Puberty" OR DE "Adolescent Development" OR DE "Adolescent Characteristics" OR DE "Adult Development"

psychotherap* OR “therap*” OR counseling OR "counselling" OR "psychological treatment*" OR "psychosocial treatment" OR "psychological intervention*" OR "psychosocial intervention*" OR psychoeducation OR "group therap*" OR "family therap*" OR “general psychiatric management” OR GPM OR MA "Mind-Body Therapies” OR "supportive psychotherapy"OR DE "Psychotherapy" OR DE "Counseling" OR DE "Psychoeducation" OR DE "Psychosocial Readjustment" OR DE "Psychotherapeutic" OR DE "Group Psychotherapy" OR DE "Family Therapy" OR DE "Creative Arts Therapy" OR DE "Mind BodyTherapy" OR DE "Dance Therapy" OR DE "Art Therapy" OR DE "Play Therapy" OR DE "Supportive Psychotherapy"

“cbt” OR "cognitive therapy" OR "behavior therapies" OR "behavior therapy" OR "behavioural treatment" OR "behavioral treatment" OR "behavioral activation" OR "exposure and response prevention" OR "exposure with response prevention" OR (exposure AND "response prevention") OR REBT OR "problem solving therapy" OR "interpersonal therapy" OR "mindfulness" OR psychodynamic OR "psychodynamic therapy" OR DE psychoanalysis OR "psychoanalysis" OR "psychoanalytic" OR "mentalization based therapy" OR "mentalization based treatment" OR "MBT" OR “transference focused therapy” OR "transference focused psychotherapy" OR “metacognitive therapy" OR "metacognitive treatment" OR "Acceptance and Commitment Therapy" OR "ACT" OR "dialectical behavior therapy" OR "dialectical behaviour therapy" OR DBT OR "Schema Therapy" OR "Schema-focused Therapy" OR "Systematic Desensitization" OR "Exposure therapy" OR MA relaxation OR "relaxation" OR DE biofeedback, psychology OR ("biofeedback" AND "psychology") OR "psychology biofeedback" OR "biofeedback") OR DE hypnosis OR "hypnosis" OR "Attention bias-modification" OR DE "Cognitive Behavior Therapy"OR DE "Behavior Therapy"OR DE "Behavioral Activation System"OR DE "Exposure Therapy"OR DE "Interpersonal Psychotherapy"OR DE "Mindfulness"OR DE "Psychodynamic OR DE Psychotherapy"OR DE "Psychodynamics"OR DE "Acceptance and Commitment Therapy"OR DE "Dialectical Behavior Therapy "OR DE "Schema Therapy "OR DE "Systematic Desensitization Therapy"OR DE "Relaxation Therapy"

(exposure AND "response prevention") OR DE "Cognitive Behavior Therapy" OR DE"Cognitive Behaviour Therapy" OR CBT OR "cognitive therap*" OR "behavior therap*" OR "behaviour therap*" OR "behavioural treatment" OR "behavioral activation" OR "exposure and response prevention" OR "exposure with response prevention OR "Acceptance and Commitment Therapy" OR "ACT" OR "dialectical behavior therapy" OR "dialectical behaviour therapy" OR DBT OR "Schema-focused therapy" OR "schema therapy" OR DE "Cognitive Behavior Therapy" OR DE "Behavior Therapy" OR DE "Behavioral Activation System" OR DE "Exposure Therapy" OR DE "Acceptance and Commitment Therapy" OR DE "Dialectical Behavior Therapy "OR DE "Schema Therapy " OR “interpersonal and social rhythm therapy” OR “IPSRT” OR DE "Interpersonal Psychotherapy" OR “cognitive training” OR “cognitive remediation” OR DE "Brain Training" OR "behavioral weight control" OR ("supportive-expressive" AND ("therapy" OR "program" OR "treatment" OR "intervention*")) OR DE „Expressive Psychotherapy“ OR DE „Supportive Psychotherapy“ OR ("insight-oriented" AND ("therapy" OR "program" OR "treatment" OR "intervention*")) OR DE „Insight Therapy“ OR "interpretive psychotherapy" OR (interpretive AND (DE psychotherapy OR ("psychotherapies" OR "psychotherapy"))) OR "General psychiatric management" OR (Systems AND (MA education OR "education" OR "training"OR "education" OR "training") AND ("MA emotions OR "emotions" OR "emotional") AND predictability AND (MA problem solving OR ("problem" AND "solving") OR "problem solving"))
